# Endocrine-responsive breast cancer: a 28-year Odyssey

**DOI:** 10.3332/ecancer.2011.237

**Published:** 2011-12-01

**Authors:** A West, KP Friedman, F Muggia

**Affiliations:** NYU School of Medicine, 550 First Avenue, New York, NY 10016, USA

## Abstract

Details on the 28-year treatment history of a patient with an endocrine-responsive breast cancer are provided. She was originally diagnosed as having a T1N0M0 cancer after a modified radical mastectomy at age 41. Fifteen years later, in 1998, she presented with hemoptysis and pleuritic chest pain: a 10 cm right atrial tumor and estrogen receptor (ER) positive endobronchial and adjacent lung parenchyma adenocarcinoma were documented. Epithelial markers normalized as she manifested a partial response (PR) lasting 3 years with tamoxifen treatment. From 2001 to 2007 she benefitted from exemestane treatment. Upon progression in the previous lung area and left adrenal, exemestane withdrawal led to transient decrease in markers. Six months later (in July 2008), with growth in her adrenal tumor, laparoscopic adrenalectomy was performed: in addition to ER positivity, the tumor showed Her2 overexpression and amplification. She has subsequently had some control of disease with fulvestrant, letrozole + trastuzumab, and subsequently letrozole + lapatinib. In addition to the chronicity of disease, this history illustrates the expanding range of treatments available for endocrine-responsive breast cancer commensurate to our greater understanding of tumor biology.

## Introduction

Hormonal modulation in patients with estrogen receptor (ER) and progesterone receptor (PR) positive metastatic breast cancer is a treatment strategy resulting in less morbidity than chemotherapy. Sequential interventions in a woman initially diagnosed at age 41 with a T1N0M0 breast cancer in 1983 and recurring in 1998 are described. This case highlights how our understanding of endocrine-based therapy and breast cancer biology offer an array of treatment options for luminal A and B subtypes of metastatic breast cancer [1].

## Case report

This patient’s treatment is best described in five parts.

**Figure 1:**
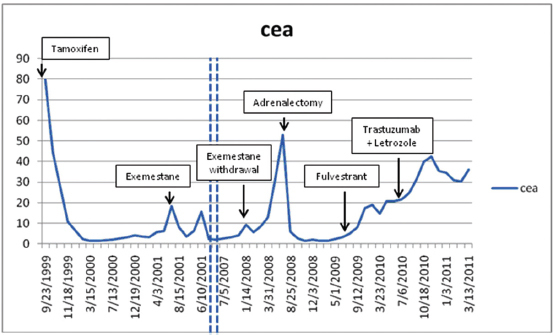
Serum CEA in relation to start of treatments.

**Figure 2:**
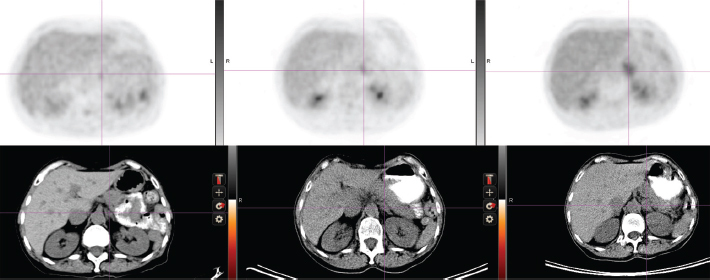
Abdominal cross-section showing left adrenal mass and FDG uptake (upper panel). See text for time points.

**Figure 3:**
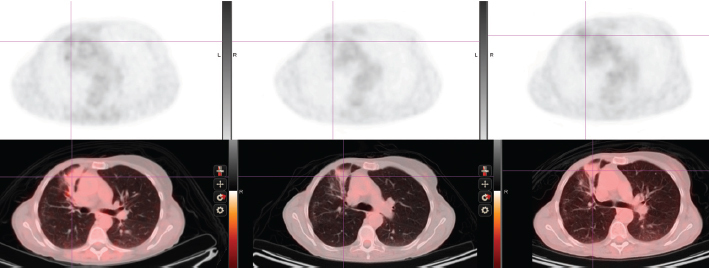
Thoracic cross-section showing right lung infiltrate and FDG uptake (upper panel). See text for time points.

- **Initial diagnosis to recurrence** (1983–1998) This woman, without a family history of cancer but of Ashkenazi Jewish origin, had routine mammography leading to a diagnosis of an invasive ductal carcinoma (T1N0M0) of the right breast at age 41. After a modified radical mastectomy and axillary node dissection (pathology and surgical report not available) she was given no adjuvant therapy. In 1998, she presented with complaints of right pleuritic chest pain, cough with hemoptysis and shortness of breath. Imaging and subsequent bronchoscopic biopsy revealed metastatic adenocarcinoma, ER and PR positive. A 10 cm lesion was noted in the right atrium. Her epithelial markers were elevated (Figure 1 summarizes the time course of CEA, and not shown, also paralleled by CA27.29).Anti-estrogen therapy (tamoxifen) was begun, with accompanying low-dose aspirin (later discontinued) to minimize the risk of thromboembolic disease.
- **Response to tamoxifen** (1998–2001) **and to exemestane** (2001–2007) Complete regression of the right atrial lesion within 6 months ensued following tamoxifen treatment. Lung findings were stable until 2001 at which time a new right lung infiltrate was noted; treatment was switched to exemestane. She experienced improvement until February 2008 when routine labs showed increasing CEA and increased ^18^fluorodeoxyglucose (FDG) uptake on PET/CT was noted in the left adrenal (Figure 2, cross section) and in lung and mediastinum (Figure 3, cross section). Both figures showing three points in time are shown: left in August 2004 in remission, middle in October 2007 progressing, and right in May 2008 prior to left adrenalectomy.
- **Brief response to exemestane withdrawal** (2008) The patient’s CEA level dropped over 2 consecutive months (Figure 1) after exemestane withdrawal to 9.2 ng/ml (1/14/08), 5.6 ng/ml (2/19/08) and 8.6 ng/ml (3/10/08). However, CEA increased to above 13 ng/ml (3/31/08), when an MRI in May 2008 noted decreased disease in the chest but a larger left adrenal mass (Figures 2 and 3).
- **Adrenalectomy for metastases followed by fulvestrant** (2008–2009) Laparoscopic removal of left adrenal tumor was achieved without incident in July 2008, as CEA dropped and remained within the normal range (<5) for 1 year. The adrenal tumor was a well-differentiated adenocarcinoma with strongly positive hormone receptors and Her2 3+ immunostaining; by FISH the Her2 had 4-fold amplification. In August 2009, as CEA began to rise, fulvestrant was started. Fulvestrant therapy was discontinued in May 2010 as CEA rose and imaging showed progression; a trial of anti-Her2 therapy combined with letrozole was instituted.
- **Endocrine therapy (letrozole) combined with anti-Her2 therapy (2010–present)** The patient eventually received letrozole and trastuzumab every 3 weeks. This treatment was discontinued on March 2011 when a surveillance PET/CT indicated a mixed response: increase in the right hilar mass, while the right upper lobe mass was smaller. Her markers have since continued to rise, accompanied by some weight loss and intermittent fevers. Additional anti-Her2 therapy including lapatinib and letrozole was started in June 2011. She and her husband continue to be reluctant to undergo trials with experimental agents; therefore, chemotherapy with capecitabine was started in August 2011 because of symptomatic progression in the lung and mediastinum.

## Discussion

Most (T1N0M0) invasive ductal carcinomas fall within the luminal A subtype [1]. Prolonged survival but with a persistent risk of recurrence at local or distant sites is typical of luminal A subtype suggesting that disease deposits may regain proliferative potential after a state of dormancy. Curiously, Her2 amplification was identified on the adrenalectomy specimen 25 years after initial diagnosis. Trial overviews have shown recurrence to be most common within 10 years of surgery; however, a significant risk of recurrence persists into the second decade [2]. The Early Breast Cancer Trialists’ Collaborative Group meta-analysis of adjuvant hormonal therapy following surgery showed a reduction in rate of recurrence and cancer death following 1–2 and 5 years of tamoxifen therapy. After 5 years of tamoxifen therapy in ER positive patients, the recurrence rates were 33.2% to 45% lower than controls at 15 years, with 25.6% mortality compared with 34.8% mortality in the control group [2].

Upon recurrence, tamoxifen was impressively effective against our patient’s metastatic disease including normalization of the epithelial markers and complete regression of the right atrial tumor and this was followed by an even longer response on aromatase inhibitors (AI)—that had by then demonstrated superior outcome compared with tamoxifen in metastatic disease [3] as well as adjuvant hormonal therapy [4]. Based on animal models, an alternating treatment regimen cycling anti-estrogen and estrogen therapy is being explored as a way to avoid resistance to hormonal agents [5]. Growth in an estrogen depleted state is accompanied by hypersensitivity to estrogens and might explain withdrawal responses that have been seen with AIs, and specifically with exemestane [6,7]. The metastasis in the left adrenal continued to enlarge during withdrawal from exemestane, while the lung infiltrate showed regression. Mixed responses may reflect heterogeneity in hormone and growth factor receptors at various sites. Discordant molecular findings (such as Her2+ amplification found in the adrenal) may be attributed to evolution in tumor biology, to sampling error due to heterogeneity including variably focal areas of receptor density, and to challenges in repeating receptor assays. Longitudinal studies in hormone and Her2 receptor status showed variations in receptor expression according to site of disease in 53 of 56 patients with metastatic breast cancer at median biopsy interval of 23.7 months. This has been interpreted as reflecting genetic instability of cancer cells, perhaps enhanced by treatment and host factors [8].

The use of surgical resection in treatment of metastatic breast cancer rather than systemic treatment is increasingly being considered in patients with so-called ‘oligometastatases’. A 2004 review in *The Breast* concluded that location greatly influences prognosis. Liver metastases are commonly seen as part of overall progression; data regarding adrenal metastasis are lacking [9].

Our patient also benefitted from other endocrine interventions, such as the ER downregulator, fulvestrant. Mechanisms leading to endocrine resistance in hormone receptor positive breast cancer are under intense study [10–12]. The combination of trastuzumab and letrozole was evaluated in a phase III clinical trial for treatment of patients with ER and Her2 positive metastatic breast cancer: lapatinib combined with letrozole for ER+, Her2 positive breast cancer was superior to letrozole alone [13]. Future directions in patients such as ours, therefore, include not only the appropriate sequences of hormonal therapies but also the introduction of molecularly targeted agents based on deeper understanding of signaling pathways.
